# Constipation

**Published:** 1879-08

**Authors:** 


CONSTIPATION

is the worst. It generally results from inattention to the calls of nature, and usually dates from childhood. When fecal matter is retained in the bowels more than twenty four hours, it commences its destructive work notably in two ways. First—Its fluid portion is absorbed and poisons the blood, causing derangement of the liver and kidneys, palpitation of the heart, dyspepsia, eructations, fetid breath, headache, and nervous debility, often verging on paralysis. Secondly—Its clayey portion, by its accumulation and impacting in the lower portion of the rectum, interrupts the circulation of the blood in the adjoining parts, and may produce, from this cause alone, piles, fistula, inflammation of the bladder, disease of the prostate gland, or cancer of the rectum. In fact, most of these diseases are developed in this way. This is a formidable array of facts, but they will have about as much effect with two-thirds of those who have these threatening evils hanging over them as moon-beams on the icebergs of the Artic Sea. From whatever cause, there is either great ignorance or great stupidity in regard to the laws of health, among a class of people who are otherwise intelligent. How many parents are there who look carefully to the habits of their children, upon which all that will make life desirable for them depends?

Cathartics and injections should not be resorted to for the relief of constipation. They always aggravate the trouble if frequently employed. Foods alone will not effect a cure in severe cases. "Kneading" the bowels has sometimes afforded relief, but, as a rule, it is not permanent We should receive a hundred letters asking us for the best remedy for constipation, if we did not mention it; and will therefore repeat what we have so often said before, that the best remedy we have ever prescribed is "Nelaton's Suppositories."

